# Supplementary material for: Multi‐dataset identification of innovative feature genes and molecular mechanisms in keratoconus
Source: J Cell Mol Med. 2024 Sep 19;28(18):e70079. doi: 10.1111/jcmm.70079 (PMC11412914; doi:10.1111/jcmm.70079)
Supplement: Supplementary file 2 — Material S2. [file JCMM-28-e70079-s002.zip › 7.GSEA analysis/5.RNF217-AS1/KEGG_GLYOXYLATE_AND_DICARBOXYLATE_METABOLISM.html]

Details for gene set KEGG\_GLYOXYLATE\_AND\_DICARBOXYLATE\_METABOLISM[GSEA]

|  || Dataset | RNF217-AS1.group.cls#h\_versus\_l |
| Phenotype | group.cls#h\_versus\_l |
| Upregulated in class | h |
| GeneSet | KEGG\_GLYOXYLATE\_AND\_DICARBOXYLATE\_METABOLISM |
| Enrichment Score (ES) | 0.29534358 |
| Normalized Enrichment Score (NES) | 0.84612125 |
| Nominal p-value | 0.6386719 |
| FDR q-value | 1.0 |
| FWER p-Value | 1.0 |
Table: GSEA Results Summary

  

Fig 1: Enrichment plot: KEGG\_GLYOXYLATE\_AND\_DICARBOXYLATE\_METABOLISM      
 Profile of the Running ES Score & Positions of GeneSet Members on the Rank Ordered List

  

| SYMBOL | TITLE | RANK IN GENE LIST | RANK METRIC SCORE | RUNNING ES | CORE ENRICHMENT || 1 | HAO1 | na | 729 | 0.482 | 0.2953 | Yes |
| 2 | GLYCTK | na | 12002 | 0.055 | 0.0069 | No |
| 3 | MTHFD2 | na | 17185 | -0.003 | -0.1401 | No |
| 4 | CS | na | 18546 | -0.016 | -0.1688 | No |
| 5 | GRHPR | na | 19117 | -0.021 | -0.1714 | No |
| 6 | PGP | na | 19504 | -0.025 | -0.1659 | No |
| 7 | HYI | na | 19635 | -0.027 | -0.1523 | No |
| 8 | MDH2 | na | 20928 | -0.039 | -0.1638 | No |
| 9 | MDH1 | na | 21164 | -0.042 | -0.1431 | No |
| 10 | ACO2 | na | 22252 | -0.053 | -0.1394 | No |
| 11 | MTHFD1 | na | 23712 | -0.071 | -0.1348 | No |
| 12 | ACO1 | na | 24031 | -0.076 | -0.0942 | No |
| 13 | AFMID | na | 26547 | -0.117 | -0.0899 | No |
| 14 | MTHFD2L | na | 27230 | -0.131 | -0.0238 | No |
| 15 | MTHFD1L | na | 27681 | -0.141 | 0.0555 | No |
| 16 | HAO2 | na | 30542 | -0.225 | 0.1206 | No |
Table: GSEA details [plain text format]

  

Fig 2: KEGG\_GLYOXYLATE\_AND\_DICARBOXYLATE\_METABOLISM      
 Blue-Pink O' Gram in the Space of the Analyzed GeneSet

  

Fig 3: KEGG\_GLYOXYLATE\_AND\_DICARBOXYLATE\_METABOLISM: Random ES distribution      
 Gene set null distribution of ES for **KEGG\_GLYOXYLATE\_AND\_DICARBOXYLATE\_METABOLISM**
